# Alpha 1 Microglobulin: A Potentially Paradoxical Anti-Oxidant Agent

**DOI:** 10.4172/2379-1764.1000238

**Published:** 2017-08-24

**Authors:** Richard A Zager

**Affiliations:** Department of Medicine, University of Washington, Seattle, WA, USA

**Keywords:** Alpha 1 microglobulin, Renal oxidant stress, Lysosomes, Heme, Myoglobin, Renal ischemia, Acute kidney injury, Acute kidney failure

## Abstract

Alpha 1 microglobulin is a low molecular weight heme binding antioxidant protein with interesting, and potentially important, clinical applications. However, much remains to be learned about its in vivo effects. This invited review raises a number of physiologic issues regarding this compound as it pertains to clinical use.

## Brief Review

Alpha 1 microglobulin (A1M) is a small globular plasma protein that is universally present in vertebrates, including humans. It is predominantly synthesized in liver and then gains systemic distribution following hepatic release. Its function is that of a potent free heme binder (second only in avidity to hemopexin) and as an antioxidant protein [1-5]. As such, there has been considerable interest in the use of recombinant A1M (rAIM) as an anti-oxidant agent, particularly in settings in which free heme or heme proteins are released into the circulation (e.g. traumatic myohemoglobinuria, intravascular hemolysis and pre-eclampsia). Given its small size (∼26 kDa; 183 amino acids), A1M is readily filtered by the renal glomerulus. Upon delivery into tubular lumina, it is largely reabsorbed by proximal tubules, presumably via the megalin-cubulin pathway [2]. Because the proximal tubule is the primary target of ischemic and nephrotoxic injuries, and because oxidant stress is evoked in virtually of forms of renal damage [6], rA1M therapy has been proposed as a renal therapeutic with potential broad based clinical application, e.g. pre-eclampsia, toxic and ischemic acute kidney injury, and chronic renal disorders such as diabetic nephropathy.

Indeed, a number of intriguing reports in the experimental literature lend support to rA1M's potential as a renal protective agent. For example, free heme toxicity is markedly suppressed with rA1M treatment (e.g. as mediated against cultured renal proximal tubule cells [7]). While multiple anti-oxidant mechanisms for A1M have been proposed (e.g. its acting as a reductase, a free radical scavenger; and protecting mitochondria by complex 1 binding) [8-15], blockade of free heme toxicity, at least *in vitro* [7] appears to largely result from direct heme binding. As such, heme is unavailable to induce its pro-oxidant effects. In addition to rA1M's *in vitro* actions, protection against experimental pre-eclampsia and radio-chemotherapy toxicity in animals has been suggested [8-12]. However, regarding its *in vivo* effects, there are some seemingly important questions as to rA1M's purported mechanism of action, particularly as a renal anti-oxidant agent. rA1M clinical trials are in the planning and execution phases (A1M Pharma; Lund, Sweden). Thus, it seems appropriate to consider a number of potential mechanistic issues in this regard:

First, it is clear that A1M has potent free heme binding effects. However, in vivo, hemopexin is a much more avid heme binder, and it is present in far greater (∼100 mg/dL) concentrations vs. A1M (∼2 mg/dl). Furthermore, albumin, the dominant plasma protein (4-5 g/dl), also serves as a free heme scavenger. Hence, it is unclear as to how rA1M could function as a quantitatively important heme binder, given the presence of far greater plasma hemopexin and albumin concentrations.

Second, given its small size (∼26 kDa) which permits rapid glomerular filtration, free rA1M has an extremely short plasma half-life (∼7 min) following its IV administration [12]. In contrast, both hemopexin (57 kDa) and albumin (67 kDa), due to their much greater sizes, are largely excluded from crossing the glomerular size-dependent perm-selectivity barrier. Thus, given stable hemopexin and albumin plasma levels, it remains uncertain as to how a transient rise in plasma rA1M could function as a quantitatively important intravascular heme scavenger.

Third, were rA1M to complex free heme within plasma, following glomerular filtration the A1M-heme complex (1:2 binding) should function as a renal proximal tubule heme delivery agent. As such, this could promote, rather than retard, oxidative damage. Indeed, an A1M-heme complex would likely mimic the nephrotoxic effects of myoglobin, another low molecular weight heme-bearing protein. In this regard, in a recent study of myohemoglobinuria, rA1M administration tended to increase, not decrease, renal oxidative stress [7]. It has recently been suggested that, following renal tubular uptake, A1M might regain access to the circulation via renal tubular transcytosis [15]. Thus, although the process of escaping lysosomal destruction might prolong A1M's biologic half-life, transcytosis might also allow for even more intravascular free heme binding, and hence, more renal heme delivery

Fourth, it is well known that free radicals act at, or close to, the site of their generation. In this regard, following proximal tubule uptake, reabsorbed proteins are specifically trafficked to lysosomes for rapid proteolytic destruction. Given this process, it is unclear as to how lysosomal-targeted and subsequently degraded, proteins could combat free radical toxicities at extra-lysosomal sites, most notably, mitochondria. Alternatively, were A1M to gain mitochondrial access during the evolution of renal injury, the potential to bind to complex 1 and mitigate oxidative injury might result [8]. However, this action is also open to at least some speculation, given that rA1M failed to mitigate oxidant induced antimycin toxicity in cultured human proximal tubule cells [7]. Alternatively, if an active A1M transcytosis pathway exists for A1M, this could potentially limit A1M mitochondrial delivery.

Fifth, it has been noted that ∼50% of native A1M circulates bound to immunoglobulins and albumin. Hence, these bound A1M forms are too large to undergo significant glomerular filtration. Hence, were rAIM to similarly complex to high molecular weight proteins, this should preclude substantial renal A1M delivery, thereby blunting a renal protective effect.

Sixth, experimental evidence exists that active endocytic uptake of low molecular weight proteins sensitizes proximal tubular cells to superimposed renal damage (e.g. such as that induced by ischemia) [16]. Furthermore, any proteins that escape proximal tubule reabsorption would be delivered to the distal tubule where they co-precipitate with Tamm Horsfall protein, forming obstructing casts. Thus, by either stimulating proximal tubule endocytosis, or by increasing distal tubule protein delivery and cast formation, potential adverse effects from low molecular weight protein (e.g. rAIM) delivery could result.

Each of the above considerations raises the question as to whether, or exactly how, administered rA1M might mediate renal protective effects. Potential pathways, above and beyond A1M's purported antioxidant activities, should be considered, e.g. possible effects on cell signaling, genomic transduction, proteolytic responses, and metabolic pathways. As just one example, this “anti-oxidant” protein, by delivering a low molecular weight protein-heme complex to tubules, could, like myoglobin, paradoxically trigger iron-mediated oxidative stress which then activates the well-known Nrf2 cytoprotective pathway [17,18]. Clearly, this is a plausible alternative explanation that could be explored.

## Conclusion

It should be recalled that in the pharmacologic sciences, an initially expected protective action of a particular agent may occur, but upon subsequent investigations, unanticipated protective mechanisms are shown to be involved. In light of the above considerations, perhaps the same might be true of A1M. These possibilities seemingly deserve consideration as clinical trials with rA1M are soon to commence.
